# Comparative genomic analysis reveals the evolution and environmental adaptation strategies of vibrios

**DOI:** 10.1186/s12864-018-4531-2

**Published:** 2018-02-13

**Authors:** Heyu Lin, Min Yu, Xiaolei Wang, Xiao-Hua Zhang

**Affiliations:** 1 College of Marine Life Sciences, Ocean University of China, 5 Yushan Road, Qingdao, 266003 People’s Republic of China; 2 Laboratory for Marine Ecology and Environmental Science, Qingdao National Laboratory for Marine Science and Technology, Qingdao, 266071 People’s Republic of China; 3 Institute of Evolution & Marine Biodiversity, Ocean University of China, Qingdao, 266003 People’s Republic of China

**Keywords:** *Vibrio*, Comparative genomics, Phylogeny, Environmental adaptation, Gene gain/loss, Chitinase

## Abstract

**Background:**

Vibrios are among the most diverse and ecologically important marine bacteria, which have evolved many characteristics and lifestyles to occupy various niches. The relationship between genome features and environmental adaptation strategies is an essential part for understanding the ecological functions of vibrios in the marine system. The advent of complete genome sequencing technology has provided an important method of examining the genetic characteristics of vibrios on the genomic level.

**Results:**

Two *Vibrio* genomes were sequenced and found to occupy many unique orthologues families which absent from the previously genes pool of the complete genomes of vibrios. Comparative genomics analysis found vibrios encompass a steady core-genome and tremendous pan-genome with substantial gene gain and horizontal gene transfer events in the evolutionary history. Evolutionary analysis based on the core-genome tree suggested that *V. fischeri* emerged ~ 385 million years ago, along with the occurrence of cephalopods and the flourish of fish. The relatively large genomes, the high number of 16S rRNA gene copies, and the presence of R-M systems and CRISPR system help vibrios live in various marine environments. Chitin-degrading related genes are carried in nearly all the *Vibrio* genomes. The number of chitinase genes in vibrios has been extremely expanded compared to which in the most recent ancestor of the genus. The chitinase A genes were estimated to have evolved along with the genus, and have undergone significant purifying selective force to conserve the ancestral state.

**Conclusions:**

Vibrios have experienced extremely genome expansion events during their evolutionary history, allowing them to develop various functions to spread globally. Despite their close phylogenetic relationships, vibrios were found to have a tremendous pan-genome with a steady core-genome, which indicates the highly plastic genome of the genus. Additionally, the existence of various chitin-degrading related genes and the expansion of chitinase A in the genus demonstrate the importance of the chitin utilization for vibrios. Defensive systems in the *Vibrio* genomes may protect them from the invasion of external DNA. These genomic features investigated here provide a better knowledge of how the evolutionary process has forged *Vibrio* genomes to occupy various niches.

**Electronic supplementary material:**

The online version of this article (10.1186/s12864-018-4531-2) contains supplementary material, which is available to authorized users.

## Background

Vibrios represent a large subgroup of the class *Gammaproteobacteria* and are Gram-negative, usually motile rods. Generally, members of the *Vibrio* species are mesophilic and chemoorganotrophic, and have a facultatively fermentative metabolism [1]. Vibrios are ubiquitously distributed in water columns of marine and estuarine water, marine sediments, and aquaculture settings worldwide [2]. It has been shown that vibrios are characterized by remarkable biodiversity, having evolved to develop complex lifestyles [3]. Many studies have indicated that vibrios have enormous biomass in the ocean, and comprises more than 10% of the aquatic culturable bacterial population [4–7]. To date, more than 110 species are recognized in the genus *Vibrio*, many of which have been identified in recent years.

As representative obligate heterotrophs, vibrios utilize a wide range of carbon sources for energy [8], an interesting feature of which is the utilization of chitin. Chitin is the most abundant polysaccharide in the ocean, and the second most abundant polysaccharide in nature after cellulose [9]. Chitin is composed of repeating units of the monomer *N*-acetylglucosamine (GlcNAc), and this material is an abundant carbon-nitrogen-energy source for marine microorganisms. Cycling of chitin is mainly by chitinolytic bacteria [10]. Many *Vibrio* species, such as *V. cholerae* [10], *V. harveyi* [11] and *V. furnissii* [12], are able to use chitin as their sole carbon source. A group of enzymes is required for vibrios to successfully convert chitin to monomer [10], in which process chitinases function to hydrolyze chitin into small oligomers. Chitinases have been received much attention and were believed to play a central role in the chitin degrading processes by bacteria [13]. Many physiological characteristics of vibrios were associated with the capacity of chitin-degradation, including chemotaxis, cell division, biofilm formation and natural competence [14].

The genomic features of vibrios in the past decades have been well studied, and with the increasing number of sequenced species, there are growing number of reports which focus on the genus rather than on specific members of the genus. The diversity and phylogenetics of vibrios has long been a matter of interest, and multilocus sequence analysis (MLSA) and constructing supertrees using housekeeping genes from whole genome sequencings has been used in the analysis [15], which was believed to be the most powerful tool until alternative genome-based methods were developed. Sawabe et al. [16] have studied the evolutionary history of vibrios using 8 housekeeping genes, and suggested the method to be very efficient and reliable. While comprising a considerable pan-genome [15, 17], the genome plasticity of vibrios aided by horizontal gene transfer (HGT) was considered to be an important method of acquiring new genes and accelerating evolution [18], and this rapid change in the composition of the genomes mainly related to cell envelope modification and secondary metabolites, helping vibrios to succeed in competition with other organisms [19]. The wide range of metabolisms in vibrios, which facilitates them to adapt to a wide array of environmental conditions has drawn much attention, especially the capability of degrading chitin [8, 12]. Nevertheless, although many studies mentioned above have been carried out to investigate the diversity, evolution and ecology of vibrios, many fundamental works are still needed in order to discover the underlying genetic basis, and this is now possible as the novel genomes and productive tools have recently become available.

For a long time, draft genomes have been employed to perform genomic studies. However, a complete sequence is better than partial genomes to examine all the genes or study the complete hereditary information of an organism [20]. The advent of the improved sensitivity and precision of complete genome sequencing technology provides a primary important path toward understanding the genome features of organisms. Lukjancenko et al. [17] noticed that the core gene numbers of *Vibrio* chromosome in complete genomes dropped sharply to only half of that when considering draft genomes, which was partly due to the incomplete information stored in the draft genomes. Besides, the rRNA operon number of draft *Vibrio* genomes is only one in most cases, whereas the complete genomes have seven or eight copies generally. Given the increased availability of complete genome sequences in the public databases and the development of comparative genomic analysis approaches, a more integrated and comprehensive view of the genus *Vibrio* becomes possible. There were more than 1500 genomes of vibrios available in the GenBank database at the time of this study, 18 of which were complete genomes sequences of the *Vibrio* species.

In this study, we sequenced two new complete *Vibrio* genomes, and presented a broad comparative analysis of these genomes across the genus with 18 other complete genomes of *Vibrio* spp. available in the GenBank database (http://www.ncbi.nlm.nih.gov). Firstly, the phylogeny of the genus *Vibrio* was inferred using core-genome. Secondly, the ancestral reconstruction and the estimation of emerge time of vibrios were performed to provide new knowledge about the evolutionary history of the genus. Thirdly, various environmental adaptation strategies, especially the ability of the chitin metabolism, were investigated to help understand how vibrios adapt to a variety of habitat niches. Finally, all the *Vibrio* genomes, including those draft sequences in the public database, along with a set of other representative marine bacteria, were employed to provide a comprehensive assessment of genome features. Our analysis helps in the understanding of the genetic characteristics of *Vibrio* and serves as a guide for further research.

## Methods

### Bacterial strains

The two strains *V. rotiferianus* B64D1 and *V. mediterranei* QT6D1 were isolated from bottom seawater of the Bohai Sea (119.04°E, 38.23°N, 17.5 m water depth) in August 2015 and the East China Sea (122.0051°E, 26.8957°N, 92 m water depth) in October 2015, respectively. No animal ethics approval was required, and the field sampling procedures met local guidelines. Both strains were isolated using thiosulfate citrate bile salts sucrose (TCBS) agar (Hopebio, China) and demonstrated strong capacity to degrade chitin when growing on chitin agar plates. The strains were routinely grown aerobically on marine 2216E agar (MA; Becton Dickinson) at 28 °C. Genomic DNA of the two strains were extracted using the phenol-chloroform-isoamylic alcohol extraction protocol described by Marmur [21], and the 16S rRNA genes were sequenced to validate the strains.

### Genome sequencing and annotation

Whole-genome shotgun sequencing of *V. rotiferianus* B64D1 and *V. mediterranei* QT6D1 was carried out with paired-end sequencing by HiSeq 4000 (Illumina) and long sequencing by PacBio RS II Sequencer (Pacific Biosciences, Menlo Park, CA). The long sequence was assembled using Canu v1.1 [22], and Pilon v1.16 [23] was used to polish PacBio assemblies according to Illumina data. Misassemblies were identified and corrected using REAPR [24] and manual inspection. Open reading frame (ORF) prediction and annotation were carried out using the RASTtk pipeline [25]. tRNA-encoding genes and rRNA operons were found by using tRNAscan [26] and RNAmmer [27], respectively. Restriction-modification (R-M) systems were identified by searching the RAST annotation, and CRISPRfinder [28] was used to search for CRISPR (clustered regularly interspaced short palindromic repeats) regions. When the CRISPR regions was recognized as questionable CRISPRs, the presence of Cas (CRISPR-associated protein) genes was considered evidence for a functional CRISPR system.

The two complete genomes sequenced in this study and a further 18 complete genomes of representative strains in distinct species of the genus *Vibrio* were collected together as the “small dataset” (Additional file 1: Table S1), while all the 1582 *Vibrio* genomes available in GenBank except those with low qualities (Contig N50 < 10,000 bp) were retrieved to make up the “large dataset”. *Vibrio* species included in the small dataset were isolated from various sources, such as oceans, estuarine environments, humans, marine fishes and marine molluscs, which enabled the provision of a holistic profile of the genus. In particular, *V. fischeri* (*Allivibrio fischeri*) and *V. damselae* (*Photobacterium damselae* subsp. *damselae*) had been considered involved in the genus *Vibrio* for a long time. Although they have been reclassified as other closely related genus [29, 30], we decided to involve them in this study to acquire richer information on the evolution and forces for genome changes of the group due to their relatively unique living strategies.

### Pan/core genomes and phylogenetic analysis

The program GET_HOMOLOGUES v3.0.3 [31], with three popular clustering algorithms, i.e. bidirectional best hit (BDBH), COGtriangles [32] and OrthoMCL [33], was used for clustering orthologous genes and identifying core- and pan-genomes within the small dataset mentioned above under default parameter values. Sequences of all the single-copy orthologues were aligned individually using the MAFFT software with the “linsi” algorithm [34], and the alignments were concatenated into one extended alignment. Subsequently, TrimAl v1.2 [35] was applied to remove ambiguous and unreliable fragments from the concatenated alignment with the parameter “-gt 1”. The phylogenetic relationship of the concatenated data was inferred using RAxMLv8.2.4 [36] based on the Maximum-likelihood (ML) algorithm with the PROTGAMMALGF evolutionary model and 1000 bootstrap replicates, after Prottest 3.4 [37] was performed to estimate the best-fit substitution model. The genome of *Shewanella denitrificans* OS217 was chosen to serve as an outgroup. The phylogenetic network of the concatenated data was constructed using SplitsTree v4.14.6, with a neighbor net drawing and Jukes-Cantor correction [38]. A pan-genome tree was constructed according to the presence/absence of all homologous genes using RAxML. Besides, we identified the specific genome (unique gene pool of a species) of the two new strains we sequenced in this study among the noncore region, in order to investigate the unique properties of them.

A chronogram for 23 taxa RAxML phylogeny was constructed using the method of penalized likelihood [39], implemented in r8s v1.81 [40]. The estimated origin time of cyanobacteria (2700~ 3500 million years ago, MYA) [41] and the divergence time between *Escherichia coli* and *Salmonella enterica* (140 MYA) [42] were used as calibration points. The reference ML tree was built based on 332 single-copy core orthologues shared among all the 23 bacterial species. The optimal smoothing parameter was determined through a cross-validation procedure that involved pruning terminal branches.

### Reconstruction of gene gain and loss events

Reconstruction of ancestral gene content and the gene gain and loss during the evolution of the small dataset was conducted using the phylogenetic gain–loss-duplication model employed in the program COUNT [43]. The number of paralogs in each genome for all the identified gene families was taken into account in the reconstruction, and the topology of the core-genome tree was used as the reference tree. All the genes acquired or lost during the evolutionary process of the genus *Vibrio* were categorized based on searching against the COG database (http://www.ncbi.nih.gov/COG/) using RPS-BLAST program. SIGI-HMM [44] was used to infer whether genes in the genomes were acquired through HGT.

### Average nucleotide identity (ANI) calculation

ANI between any two genomes of the small dataset was calculated using a custom Perl script adapted from ANI.pl (https://github.com/jhbadger/scripts/blob/master/ANI.pl), following the algorithm described by Goris et al. [45], and the resulted matrix was clustered and visualized using R packages pheatmap software [46].

### Analysis of genes related to chitin degradation

Functional proteins related to chitin metabolism in all annotated genomes were determined according to the chitin pathway defined for *V. cholerae* [8] and by being searched against all the annotations. Among these predicted chitinolytic enzymes, the evolutionary history of chitinase A in the small dataset was inferred using the Neighbor-Joining method [47] implemented in MEGA7 [48], and the phylogenetic tree was further tested for the selection pressure among amino acid sites using several codon-based ML procedures as implemented in the codeml tool of PAML v4.9 [49]. The conserved domains of chitinase A were defined by the NCBI batch CD-search online tool (https://www.ncbi.nlm.nih.gov/Structure/bwrpsb/bwrpsb.cgi).

### Genomic comparison with other marine bacteria

Thirteen complete genomes of common marine bacteria (Additional file 2: Table S2) were retrieved from GenBank to make comparisons with *Vibrio* genomes. These genomes are from four different classes including *Cyanobacteria*, *Alphaproteobacteria*, *Gammaproteobacteria*, and *Flavobacteria*, which are considered to account for up to 80% of the total bacterioplankton in the ocean [50]. Basic features of these 13 genomes, including genome sizes, 16S rRNA gene copy numbers, the number of genes derived from HGT process and genes encoding chitinase, were inferred according to the same methods as for the *Vibrio* species described above.

### Database accession numbers

The complete genome sequences of *V. rotiferianus* B64D1 and *V. mediterranei* QT6D1 have been deposited in NCBI GenBank server under the accession number CP018311 to CP018312 and CP018308 to CP018310, respectively.

## Results

### General features of *Vibrio* species

**Fig. 1:**
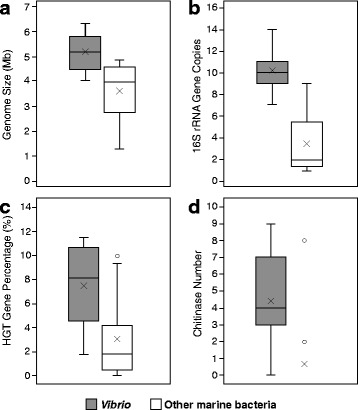
Comparison of genomic characteristics between the 20 vibrios with complete genomes and other representative marine bacteria. Box-and-whisker plot showing data distribution and interquartile ranges for each group with the median value displayed as a line in each box plot. Outliers are shown as circles, and mean values are shown as saltires in the figure. **a** Comparison of the genome sizes. **b** Comparison of the copy numbers of 16S rRNA gene. **c** Comparison of the percentage of HGT genes. **d** Comparison of the number of chitinase coding genes

The general information of the 20 complete *Vibrio* genomes is summarized in Table S1. Each genome contains two chromosomes, and some species have one or more plasmids. The genome size of vibrios ranges from 4.09 to 6.32 Mb, with a mean size of 5.14 Mb. Most the other marine bacteria compared in this study have a lower genome size (3.62 Mb on average) compared to vibrios (Fig. 1a). Vibrios have a wide range of G + C content, ranging from 38.3% in *V. fischeri* to 50.7% in *V. furnissii*. The ORF number and orthologue number range from 3807 to 5920 and 3669 to 5593, respectively. Strikingly, vibrios contain a much higher number of 16S rRNA gene copies (7–15) than most of the other marine bacteria (3 on average) used for comparison in our study (Fig. 1b), which may reflect the capability of rapid growth of *Vibrio* species. In addition, the genome size of the large dataset ranges from 2.9 to 6.7 Mb, and the G + C content ranges from 38.0% to 57.2%, which suggests a higher diversity of *Vibrio* genomes. However, due to the missing information of those draft genomes, there is some bias definitely existing in the large dataset.

### R-M and CRISPR systems

Microbes rely on diverse defense mechanisms that protect them from mobile elements or foreign DNA implicated in the reduction of HGT. The common bacterial defense systems include R-M systems and CRISPR systems [51]. We identified these two defense systems in the small dataset (Additional file 1: Table S1), and found that almost all the *Vibrio* genomes contained the R-M system except for *V. breoganii* FF50 and *V. tritonius* JCM 16456. Specifically, all the vibrios containing R-M systems have type I R-M system, while type II R-M system was additionally harbored by *V. cholerae* KW3, *V. rotiferianus* B64D1, *V. tubiashii* ATCC 19109 and *V. vulnificus* FORC 017, and type III R-M system was additionally harbored by *V. coralliilyticus* RE98. Eight out of the 20 genomes had CRISPR systems (Additional file 1: Table S1), while the cas gene numbers (1 to 8) and the lengths of direct repeats (DRs) of each CRISPR (23 to 51) were distinct from each genome. The identification of DRs in the CRISPR would be quite difficult if performed in draft genomes, since the short and repetitive sequencing reads would be difficult to assembly correctly.

### Core and pan-genome

To determine the global gene repertoire and the genes conserved across all the vibrios, the power law model of Tettelin et al. [52] was used to describe the pan- and core-genomes based on the 20 complete *Vibrio* genomes. All the genes were grouped into 21,844 families, which represented the *Vibrio* pan-genome. The progression of the pan- and core-genomes after ten random samplings was calculated (Additional file 3: Figure S1). The number of novel gene families in the pan-genome gradually increased with no limitation when more genomes were considered, leading to an open pan-genome. Nevertheless, the number of conserved gene families constituting the core genome decreased slightly, and the extrapolation of the curve indicated that the core genome reached a minimum of 1630, and would remain relatively constant, even as many more genomes were added.

### Specific genomes of V. rotiferianus B64D1 and V. mediterranei QT6D1

To investigate the unique characters of the two complete genomes sequenced by us, we identified the specific genome of each genome, i.e., orthologous families that contain genes exist in each of these two genomes but absent in other 18 previously sequenced genomes. In the total 21,844 pan-genome families, 397 specific genes were possessed by *V. rotiferianus* B64D1, whereas 871 were possessed by *V. mediterranei* QT6D1. The COG functional annotation of the specific genomes (Additional file 4: Figure S2) showed that genes related to carbohydrate transport and metabolism (G) and transcription (K) are extremely abundant (53 and 40, respectively) in *V. mediterranei* QT6D1, while the most abundant COG category in the specific genome of *V. rotiferianus* B64D1 is general function prediction only (R) and cell wall/membrane/envelope biogenesis (M). Furthermore, it is notable that there is a chitinase gene (BSZ05_18380) annotated by the RAST server involved in the specific genome of *V. mediterranei* QT6D1, which may contribute to its strong degradation capability for chitin.

### Phylogenetic and divergence times analysis of vibrio species

**Fig. 2:**
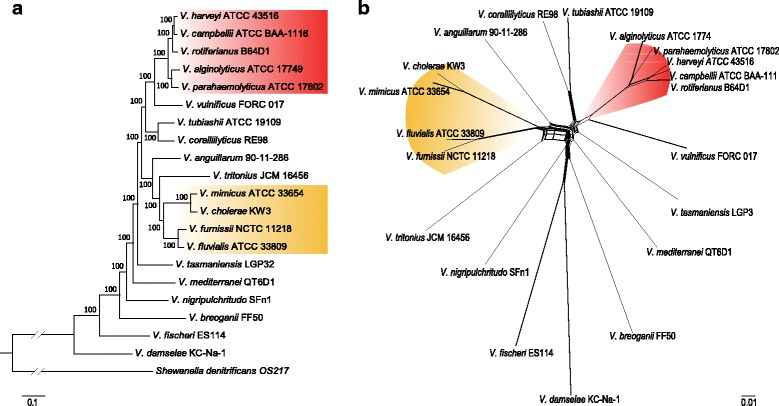
Phylogenetic relationships of the 20 vibrios with complete genomes. The Harveyi clade and the Cholerae clade are highlighted in red and yellow respectively. **a** Core-genome tree reconstructed by RAxML software. The tree is rooted using *S. denitrificans* OS217. The number at each node denotes the bootstrap value based on 1000 replicates, and the scale bar indicates the number of substitutions per site. **b** Phylogenetic network reconstructed by SplitsTree4 software

To investigate the phylogenetic relationship of *Vibrio* species, a robust core-genome tree was inferred based on the concatenated 1003 single-copy orthologues proteins shared by all the 20 complete genomes as shown in Fig. 2a. Two main clades could be determined in the tree, and these two clades could be further proved by the split network analysis (Fig. 2b). Clade I consisted of five species: *V. harveyi*, *V. campbellii*, *V. rotiferianus*, *V. alginolyticus* and *V. parahaemolyticus*, which was the so-called *Vibrio* core group [53] or Harveyi clade, and Clade II consisted of *V. cholerae*, *V. mimicus*, *V. fluvialis* and *V. furnissii*, which formed the Cholerae clade proposed by Sawabe et al. [54]. The pan-genome tree (Additional file 5: Figure S3) also had a relatively high bootstrap values at most of the nodes similar to the core-genome tree. However, the topologies of the pan- and core-genome trees were different, which might be attributed to the dispensable genes. The phylogenetic tree based on 16S rRNA genes was also inferred (Additional file 6: Figure S4), whereas it was not as reliable as the core-genome tree insofar as the bootstrap values were extremely low.

**Fig. 3:**
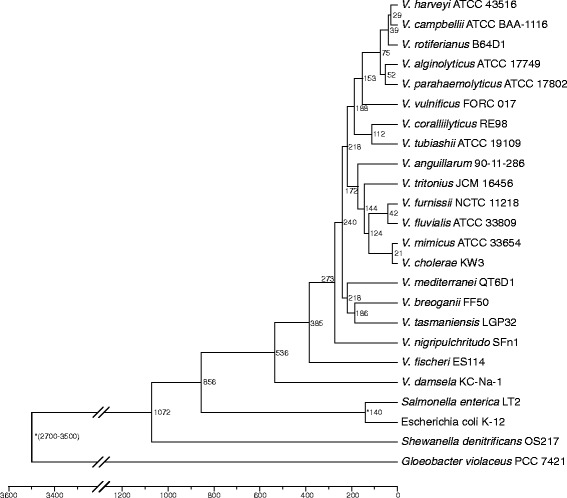
Chronogram of the 20 vibrios with complete genomes. The value near each internal branch is the estimated emerge time for that branch. Nodes with fossil record corrections are indicated with an asterisk

To explore the divergence times of vibrios, several calibration points were included in the analysis. The most recent common ancestor (MRCA) of vibrios (i.e., the emergence time of *V. damsela*) was estimated to have emerged around 536 MYA, whereas *V. fischeri* was estimated to have emerged around 385 MYA (Fig. 3).

### ANI values

ANI values calculated through pairwise comparison of the 20 *Vibrio* genomes further elucidated their genetic relatedness. ANI has been considered the best approach to measure genetic distance between genomes, as it represents a mean of identity/similarity values between homologous genomic regions shared by two genomes, which enables minimization of bias brought about by variable evolutionary rates and HGT events [55]. It was demonstrated that ANI values within a genus were generally higher than 62%, and 95–96% ANI has been recommended as the delimitation criterion for different species [56]. The ANI values between *Vibrio* species ranged from 70% to 88%, which was within the range of the classical boundary for a genus. Species groups could be derived after clustering according to the rows (Additional file 7: Figure S5), among which the Harveyi clade and Cholerae clade were consistent with the core-genome tree (Fig. 2a). Members of the Harveyi clade shared the highest ANI across the genus ranging from 80% to 88%, indicating extremely close genetic relatedness within the Harveyi clade. Furthermore, the clades possessing high ANI values could serve as an explanation for the relatively short terminal branch lengths of their members in the phylogenetic tree, as the ANI represented the genetic distances between genomes.

### Gene gain and loss

**Fig. 4:**
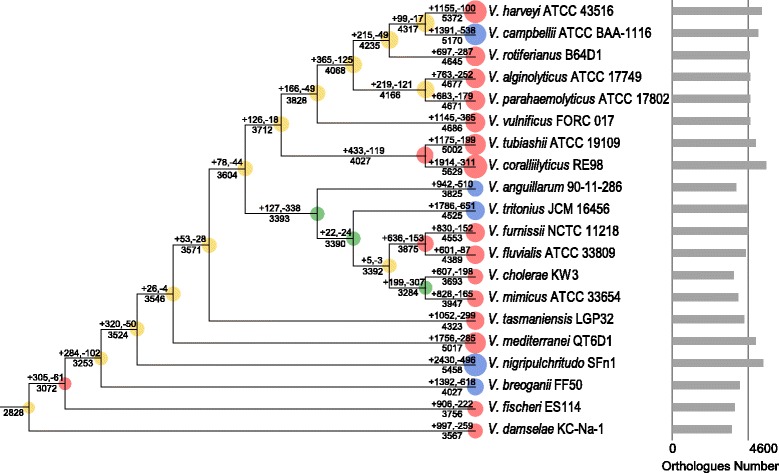
Ancestral genome content reconstruction of the 20 vibrios with complete genomes. The numbers of gene gain and loss are shown above each branch, and the numbers of gene present at each node are shown below the corresponding branch. The area of the circle at each node indicate the genome size of the node. Nodes with more gene gain events (net gains > net losses) are indicated in yellow; Nodes with more gene loss events (net losses > net gains) are indicated in green; Nodes with significant genome expansions (> 10% net gains) are indicated in red; Nodes with significant genome expansions and reductions at the same time (> 10% net gains and > 10% net losses) are indicated in blue. The bar chat on the right indicates the orthologues number of each terminal node

**Fig. 5:**
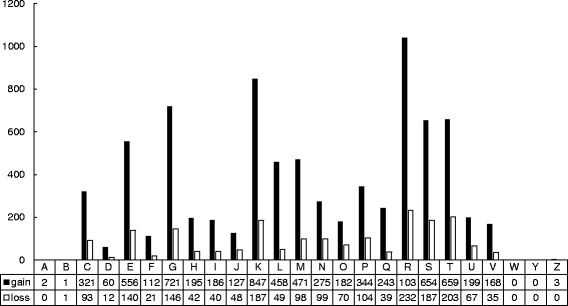
COGs classification of putative gene gains and losses occurred in the evolutionary history of vibrios**.** Designations of functional categories: [A] RNA processing and modification, [B] chromatin structure and dynamics, [C] energy production and conversion, [D] cell cycle control and mitosis, [E] amino acid metabolism and transport, [F] nucleotide metabolism and transport, [G] carbohydrate metabolism and transport, [H] coenzyme metabolism, [I] lipid metabolism, [J] translation, [K] transcription, [L] replication and repair, [M] cell wall/membrane/envelope biogenesis, [N] Cell motility, [O] post-translational modification, protein turnover, chaperone functions, [P] Inorganic ion transport and metabolism, [Q] secondary metabolites biosynthesis, transport and catabolism, [R] general functional prediction only, [S] function unknown, [T] signal transduction, [U] intracellular trafficking and secretion, [V] Defense mechanisms, [W] Extracellular structures, [Y] nuclear structure, [Z] cytoskeleton

To gain insight into the origin and the variations of *Vibrio* genomes, we investigated the gene gain/loss events that might have occurred during the evolution of the genus *Vibrio*. The gene family numbers showed a noticeable upward trend during the evolutionary history, and the first time that gene loss events exceeded gene gain events was present at the MRCA of *V. anguillarum* 90–11-286 and *V. mimicus* ATCC 33654 (Fig. 4). This internal node led to a cluster that was mainly comprised by the Cholerae clade, and all the members at terminal nodes in this cluster had relatively small orthologues families (< 4600), which may be explained by the fact that gene loss events posed more significant impact on this cluster in the evolutionary process. On the contrary, other members in this phylogenetic tree experienced more and continuous gene gain events. However, no matter whether the species have suffered more gene loss events in a few internal nodes, the gene gain events in the evolutionary process were more numerous than the gene loss events in most instances, suggesting that vibrios have experienced remarkable gene family expansion in the evolutionary history. This generally expansive trend made the orthologue number of vibrios increase by approximately 50%, i.e., from 2828 gene families in MRCA of vibrios to 4547 gene families on average as currently predicted (Additional file 1: Table S1). According to the COG functional annotation (Fig. 5) of the gain/loss genes, other than the poorly characterized genes (general function prediction only and function unknown, COG categories R and S), genes related to transcription (K) formed the most abundant category of gene gains, followed by genes involved in carbohydrate transport and metabolism (G), whereas the most abundant category of gene losses was associated with signal transduction mechanisms (T). Gene gains were present in every COG category except for extracellular structures (W) and nuclear structure (Y, not applicable to prokaryotic COGs), whereas gene losses in the history were not present in RNA processing and modification (A), extracellular structures (W), nuclear structure (Y) and cytoskeleton (Z, not applicable to prokaryotic COGs).

HGT is a significant driver of gene innovation in the life of bacteria [57]. A search for genes with aberrant codon usage statistics was carried out to predict possible genomic islands, referred to as alien genes. Surprisingly, the average percentage of alien genes among these vibrios was predicted to be 7.3%, with some species containing up to 10% alien genes. The class *Gammaproteobacteria* was the major donor of HGTs, contributing as much as 72.1% alien genes, followed by *Sphingobacteria*, which contributed 6.5% of the alien genes. This percentage of alien genes was significantly higher than that of the other compared marine bacteria, with nearly all of the genomes only involved having no more than 5% alien genes, except for species from *Marinobacter* and *Rhodobacter*, which had 9.96% and 9.25% alien genes, respectively (Fig. 1c).

### Chitin degradation related genes

Many *Vibrio* species have been reported as capable of degrading chitin. Chitin degradation is achieved by a complex pathway including multiple chitinases [58]. Seventeen different protein families related to chitin metabolism were retrieved from *Vibrio* genomes (Additional file 8: Figure S6). Eighteen out of the 20 vibrios (except for *V. breoganii* FF50 and *V. tritonius* JCM 16456) contained chitinase (EC 3.2.1.14), and all the 20 vibrios contain β-N-acetylhexosaminidase (EC 3.2.1.52). Moreover, ~ 99% of the genomes in the large dataset carried both these two genes, which have the ability to completely hydrolyze chitin to monomer GlcNAc [59]. In contrast to this abundance, these genes seldom exist in the compared marine bacteria, except for strains from genus *Pseudoalteromonas* and *Shewanella*, which carried eight and two chitinase encoding genes in their genomes, respectively (Fig. 1d).

**Fig. 6:**
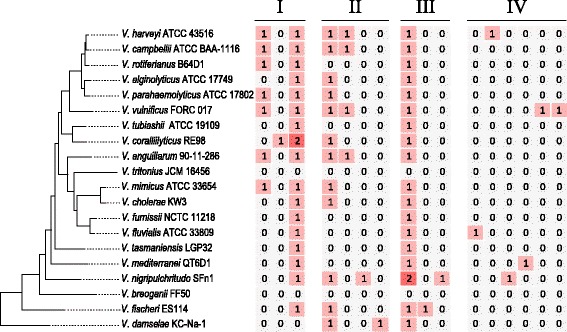
Distribution of Subgroups of enzyme Chitinase involved in the 20 *Vibrio* species with complete genomes. Each of the columns corresponds to a chitinase cluster. The number within the box indicates the copy numbers of that gene cluster. Class I contains a family 18 chitinase domain; Class II contains a family 18 chitinase domain with a chitinase insertion domain; Class III contains a family 19 chitinase domain; Class IV contains no significant chitinase domain based on NCBI conserved domain database. Columns belonged to the same class indicate they share similar component patterns of structure domains but failed to be clustered into the same protein family

**Fig. 7:**
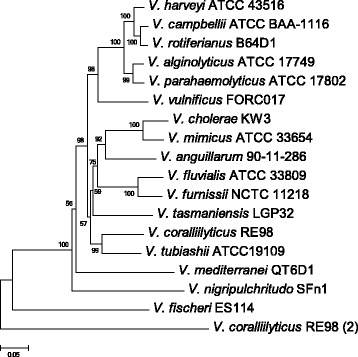
Phylogenetic tree built based on *chiA* carried by 17 complete genomes of *Vibrio*. The phylogenetic tree was obtained using the neighbor-joining method. The number at each node denotes the bootstrap value based on 1000 replicates. The ‘*V. coralliilyticus* RE98 (2)’ branch represents the second copy of *chiA* involved in strain *V. colralliilyticus* RE98

Chitinases distributed in these 18 *Vibrio* species come from 15 protein families, which can be classified into four categories (Fig. 6). Class I encompasses a family GH18 chitinase domain, while Class II possesses a similar chitinase domain as Class I but with an insertion domain in it. Class III encompasses a family GH19 chitinase domain, which was considered to be rare in bacteria [60]. However, all the 18 *Vibrio* genomes carried GH19 chitinase according to our analysis, indicating that GH19 chitinase is abundant in vibrios. Class IV does not contain any typical chitinase domain, but could be annotated as chitinase using blastp against the nr database (ftp://ftp.ncbi.nlm.nih.gov/blast/db/), which may result from the relatively low conservation of these chitinases. Most studies on diversity of chitin-degrading enzymes in aquatic environments focused on chitinase A, which belonged to Class I chitinase mentioned above, since this enzyme was thought to be conserved in Proteobacteria and may be a potential indicator of chitinoclastic ability [8]. Gene *chiA* was present in 17 species of the genus *Vibrio*, almost all of which were single-copy, except *V. coralliilyticus*, which has two different copies of *chiA*. The phylogenetic tree of 18 *chiA* genes was constructed as shown in Fig. 7. The topology of the *chiA* tree was very similar to that of the core-genome tree (Fig. 2a), indicating the *chiA* gene evolved along with the *Vibrio* genus. Intriguingly, one of the two copies of *V. coralliilyticus* was shown to have a relatively distant relationship with other orthologues, suggesting its different evolutionary origin. Furthermore, there were more synonymous (Ks) changes than non-synonymous (Ka) changes in the evolutionary process of *chiA*, which indicated the *chiA* gene had been subjected to significant purifying (negative) selection.

## Discussion

### Genomic characters of V. rotiferianus B64D1 and V. mediterranei QT6D1

The specific genome of an organism among a related group represent its unique character. We identified the specific genome of each of the two complete genomes we sequenced and found they occupied many unique orthologues families which absent from the previously genes pool of the complete genomes of vibrios. *V. rotiferianus* B64D1 comprised many unique genes related to cell wall/membrane/envelope biogenesis (COG category M), e.g., six orthologues families were annotated as glycosyltransferase, which may play an important role as the first level of protection against environmental stress. Up to 53 orthologues families in the specific genome of *V. mediterranei* QT6D1 were assigned into the COG category of carbohydrate transport and metabolism (G), among which 6 orthologues families were annotated as the component of ABC-type sugar transport system, which may indicate the potential specific ability for *V. mediterranei* QT6D1 to utilize different kinds of sugar in ambient environments. Besides, 46 orthologues families in the specific genome of *V. mediterranei* QT6D1 were assigned into the COG category of transcription (K), and 31 out of these 46 families were transcriptional regulator. The abundant unique transcriptional regulators present in the genome of *V. mediterranei* QT6D1 could enable it to regulate metabolic processes to adapt complex habitats. Furthermore, it is interesting that *V. mediterranei* QT6D1 carried a putative chitinase gene in its specific genome, which might explain the high activity to degrade chitin showed by the strain in our chitin degradation study.

### Genomics and the evolutionary dynamics of *Vibrio* species

The genus *Vibrio* encompasses one of the most diverse group of heterotrophic marine bacteria [61, 62]. We investigated 20 *Vibrio* species with complete genomes (18 species were from the GenBank and two were from this study) and all the 1582 available draft *Vibrio* genomes in the GenBank. A set of common marine bacterial species which are widely distributed in the ocean was employed to make comparisons with vibrios. The numbers of predicted ORFs in *Vibrio* species differed from ~ 3800 to ~ 6000. Given the close phylogenetic relationship of these bacteria according to the ANI analysis, such a difference suggested substantial gene loss and/or gain in their evolution. Variable gene composition was the primary mode of adaptive evolution in microorganisms, leading to the enhancement of their physiological and ecological capabilities [63]. We therefore performed ancestral state reconstruction of vibrios and found generally expansion of the orthologues number throughout the whole evolutionary history of this group. Genome expansion evidence has been found in a few other groups of marine bacterioplankton, such as the genus *Glaciecola* [64], whereas the genome reduction (also known as the genome streamlining) has been reported in groups such as SAR11 [65] and the genus *Prochlorococcus* [66]*.* Many putative gene gains belonged to the function of transcription and carbohydrate transport and metabolism, which enabled vibrios to quickly response to environmental changes and use many different kinds of substrates to survive under various conditions. The function of signal transduction mechanisms has experienced the most gene loss, despite it has also experienced more than three times of gene gain in the evolution. The constantly changing of the components of this function may indicate vibrios absorb and discard genes as needed, in response to their environments. The massive genome content changes helped vibrios succeed within a niche, and the process of HGT can promote their evolution. We found that the percentage of alien genes in *Vibrio* genomes was significantly more than most of the other compared marine bacteria, suggesting that alien genes provided vibrios with a genetic basis of diversity and helped them to survive under their respective niche conditions. In addition, the HGT events appear to occur among bacteria in close relationship, as more than 70% alien genes of vibrios were estimated to come from the same class *Gammaproteobacteria.*

Bacteria have to change their genomes to adapt to variable conditions, and greater niche diversity will require larger pan-genomes. Given the varying marine environments in which the species distributed, it was understandable that vibrios had a large amount of pan-genome contents, and this feature was in accordance with previous reports of some other marine bacteria such as the genus *Shewanella* [67] and *Glaciecola* [64]. In addition, the pan-genome tree was inferred based on homologous genes distributed in the whole-genome scales. The taxa relationships in the pan-genome tree differed from that in the core-genome tree, because the pan-genome tree focused on those genes that were not present in every genome, while on the contrary the core-genome tree focused on those genes that were present in all the genomes. Such a substantial difference between the two trees indicated that the dispensable genes, which may contain genes obtain from the HGT processes, played a crucial role in vibrios’ genomic composition and evolution.

### Phylogeny and divergence times of *Vibrio* species

*Vibrio* species have considerable biodiversity and extremely close phylogenic relationships within the genus [16]. 16S rRNA gene sequences could only provide a relatively low discriminating power to elucidate the evolutionary relationship of the group. The availability of complete genomes of *Vibrio* species enables a more definitive analysis of their relationships. Though highly plastic genomes may suffer lots of recombination and HGT events, it has been proved that the phylogenetic signal was still very strong within the core-genome [68]. We constructed a core-genome tree of the genus *Vibrio* from concatenated proteins of all the homologues shared by every species, and the result showed improved resolution and enormously increased robustness of phylogenetic analyses with nearly all the nodes having the bootstrap value of 100. The availability of a robust phylogeny of the genus *Vibrio* enabled a reliable basis to conduct the following and future analysis.

The species *V. fischeri* might have occurred at 385 MYA, corresponding to the Devonian period (~ 419.2–358.9 MYA). This was a period that the cephalopods (the order which squid belong to) has been estimated to divergent [69]. Besides, fish reached substantial diversity during this time, and many ray-finned fishes (Actinopterygii) occurred during the Devonian period [70]. Based on the phylogenetic tree, *V. fischeri* was a early member of vibrios to emerge, and it is a marine luminous bacterium that lives symbiotically with squids and fish [71]. Therefore, we could deduce that the rise of vibrios might have coincided with the occurrence of cephalopods and the flourishing of fish.

### Environmental adaptation strategies of vibrios

Free-living bacteria encompass large genomes in most cases [72], and vibrios occupy relatively large genomes compared to most other common marine bacteria in this study, which enable them to search for resources (chemotaxis), change strategies to exploit patchy nutrients and target metabolites such as complex polysaccharides, which require complex enzyme repertoires.

The capacities of microorganisms to adapt to various environments can be laterally indicated by their growth rates. To the best of our knowledge, vibrios have the fastest growth rate of marine bacteria, and *V. natriegens* has been reported to have a maximal generation time of less than 10 min [73]. It has been considered that the number of rRNA operons may dictate the rate of ribosomes synthesis and the response to favorable changes in growth environments [74]. Therefore, the extremely rapid growth rate of vibrios may be explained by the much higher number of 16S rRNA gene copies, which was, on average, more than three times of other compared marine bacteria in this study.

Bacteria have developed protective systems including R-M systems and CRISPR systems to defend external DNA invasion either by plasmids or virus [51]. Most vibrios in our analysis possessed at least one type of R-M system, and few of them also had CRISPR systems. These protective systems found in *Vibrio* genomes indicate their ability to defend themselves in marine environment and also suggest multiple encounters with foreign DNA.

Chitin metabolism is essential for carbon and nitrogen recycling in marine ecosystems. The capability of vibrios to use chitin provided possibilities for them to form biofilms on the surface of chitin, and the formation of these biofilms could affect many other biological functions [75]. Therefore, the chitinase family played an essential role in the growth and development of vibrios, which may explain the expansion of this family during the evolutionary process of the genus. We found that the numbers of chitinase genes in the genomes of *Vibrio* species (up to 9 and 16 in the small and large dataset, respectively) are much higher than that in the other compared marine bacteria (only two strains from *Gammaproteobacteria* had eight and two, respectively, while others did not carry chitinase genes), yet only two of which exist in the predicted MRCA of the genus *Vibrio*. As an expansion in size of a particular gene family suggested its importance for the adaptation of the species during evolution [76], the remarkable expansion of chitinase gene demonstrated the importance of the chitin catabolism process in vibrios.

The chitinolytic cascade in *Vibrio* species was attributed to a bunch of chitin degrading related genes. We found that ~ 99% of *Vibrio* strains contained at least one chitinase coding gene in their genomes in the large dataset. However, a previous chitinase enzyme activity survey found that only ~ 80% (37 out of 47) of *Vibrio* strains had the ability to grow on chitin substrates [8], which may be because some components required in the complex pathway of chitin-degrading were absent in the genomes. Studies on the nonpathogenic marine bacterium *V. furnissii* showed that chitin utilization was a complex process involving at least three steps: chitin sensing, attachment, and degradation [77], and it has been demonstrated that the lack of essential chitin degradation elements led to the loss of chitin-degrading activity [78]. Our analysis showed that the chitinase coding genes suffered HGT and duplication during the evolutionary history of vibrios, which may result in mutations in the sequences and followed by the loss of the chitin-degrading activity. In addition, the selective pressure calculation and phylogenic analysis of the gene *chiA* showed that the gene has undergone significant purifying selective force and may evolve along with most other protein families in vibrios. It could be deduced that there has been evolutionary pressure to make *chiA* conserve the ancestral state, and members of the *chiA* family may evolve independently by silent nucleotide substitution and may be subject to evolution by a birth-and-death process at the DNA level, which implies new genes are generated by gene duplication, and some duplicate genes may maintain in the genome, while others may be deleted or dysfunction through deleterious mutations [79]. A previous study showed that vibrios tend to acquire new genetic material more easily when they attach to chitin, and this process could help vibrios to grow in a previously hostile niche by slightly changing their genome content [80]. We noticed that the chitinase genes in *Vibrio* genomes presented random distribution patterns, and does not seem to correlate with phylogenetic relatedness, which may because the HGT or duplication events occurred in the *Vibrio* evolutionary history were randomly. Intriguingly, in the 19 *Vibrio* complete genomes, the two genomes of which does not possess genes encoding chitinase coincide with the two does not have R-M systems, suggesting that the R-M systems might be deleted from the genomes as the systems would be less useful for vibrios which lose chances to intake external DNA. This confirmed the important role of chitin metabolism in vibrios, and the fact that chitinase genes were abundant in *Vibrio* genomes potentially explained the reason why we found an extremely high rate of HGT occurring in vibrios.

## Conclusions

This extensive comparative genomics study based on complete genomes revealed the evolutionary history and ecological adaptation strategies of the widely distributed marine group vibrios. The phylogenetic history of vibrios was inferred according to the core-genome tree, and the extremely gene gain events were found during the evolutionary process from 536 MYA, which may be largely driven by HGT. The steady core-genome and tremendous pan-genome made vibrios comprise necessary genetic materials and flexible accessory genes to be cosmopolitan. Most vibrios had defensive mechanisms including R-M systems and CRISPR systems to resist the invasion of foreign DNA. Furthermore, chitin-degrading related genes were carried in nearly all the *Vibrio* genomes, and the number of chitinase genes (up to 16) have extremely expanded from only two exist in the most recent ancestor of vibrios. Our study provided a comprehensive view of vibrios at genomic level, shedding light on the process that the evolution drives the abundant heterotrophic bacteria to be diversity and adapt to various marine environments.

## Additional files

Additional file 1: **Table S1.** General features of the 20 complete genomes of *Vibrio* species analyzed. (DOCX 48 kb)

Additional file 2: **Table S2.** General features of the 13 marine bacteria complete genomes used to compare with *Vibrio*. (DOCX 34 kb)

Additional file 3: **Figure S1.** Pan and core plot of the 20 *Vibrio* species with complete genomes. Accumulation curves for (A) the number of genes in common or (B) total number of genes are plotted. The grey dots represent 10 different random input sequences of genomes, and the extrapolated limiting value for core genes is shown as a dashed line. Equations which can be used to fit the curves are shown above the plot respectively. (PDF 1023 kb)

Additional file 4: **Figure S2.** COGs classification of the specific genomes of *V. rotiferianus* B64D1 and *V. mediterranei* QT6D1. Designations of functional categories: [A] RNA processing and modification, [B] chromatin structure and dynamics, [C] energy production and conversion, [D] cell cycle control and mitosis, [E] amino acid metabolism and transport, [F] nucleotide metabolism and transport, [G] carbohydrate metabolism and transport, [H] coenzyme metabolism, [I] lipid metabolism, [J] translation, [K] transcription, [L] replication and repair, [M] cell wall/membrane/envelope biogenesis, [N] Cell motility, [O] post-translational modification, protein turnover, chaperone functions, [P] Inorganic ion transport and metabolism, [Q] secondary metabolites biosynthesis, transport and catabolism, [R] general functional prediction only, [S] function unknown, [T] signal transduction, [U] intracellular trafficking and secretion, [V] Defense mechanisms, [W] Extracellular structures, [Y] nuclear structure, [Z] cytoskeleton. (PDF 822 kb)

Additional file 5: **Figure S3.** Pan genome tree. The tree was created based on the presence or absence of gene clusters in the 20 complete *Vibrio* genomes. The number at each node denotes the bootstrap value based on 1000 replicates. The color red and yellow are corresponding to the core genome tree, suggesting the discrepancy between core and pan genome trees. (PDF 194 kb)

Additional file 6: **Figure S4.** Neighbor-joining phylogenetic tree of the 20 vibrios genomes based on 16S rRNA gene. The number at each node denotes the bootstrap value based on 1000 replicates. *S. denitrificans* OS217 was used as the outgroup. Bar, 0.01 substitutions per site. (PDF 186 kb)

Additional file 7: **Figure S5.** Heatmap presentation of pairwise average nucleotide identity of the 19 *Vibrio* species with complete genomes. The genomes are hierarchical clustered according to the values of rows. The clusters present in blue and orange are corresponding to the core genome tree. (PDF 212 kb)

Additional file 8: **Figure S6.** Genes related to the chitin-degrading process in 20 vibrios with complete genomes. Each column indicates a chitin metabolism-related gene family, with the family name indicating the predicted function. The number in the box indicates the copy number of that gene family in the corresponding genome. (PDF 282 kb)

## Abbreviations

ANI: Average nucleotide identity
HGT: Horizontal gene transfer
MA: Marine 2216E agar
ML: Maximum-likelihood
MLSA: Multilocus sequence analysis
MYA: Million years ago
ORF: Open reading frame
TCBS: Thiosulfate citrate bile salts sucrose

## References

[1] Thompson FL, Iida T, Swings J (2004). Biodiversity of vibrios. Microbiol Mol Biol Rev.

[2] Colwell RR (1996). Global climate and infectious disease: the cholera paradigm. Science.

[3] Reen FJ, Almagro-Moreno S, Ussery D, Boyd EF (2006). The genomic code: inferring *Vibrionaceae* niche specialization. Nat Rev Microbiol.

[4] Kaneko T, Colwell RR (1973). Ecology of *Vibrio parahaemolyticus* in Chesapeake Bay. J Bacteriol.

[5] Simidu U, Taga N, Colwell RR, Schwarz JR (1980). Heterotrophic bacterial flora of the seawater from the Nansei Shoto (Ryukyo Retto) area. Nippon Suisan Gakkaishi.

[6] Eilers H, Pernthaler J, Glöckner FO, Amann R (2000). Culturability and in situ abundance of pelagic bacteria from the North Sea. Appl Environ Microbiol.

[7] Yooseph S, Nealson KH, Rusch DB, McCrow JP, Dupont CL, Kim M (2010). Genomic and functional adaptation in surface ocean planktonic prokaryotes. Nature.

[8] Hunt DE, Gevers D, Vahora NM, Polz MF (2008). Conservation of the chitin utilization pathway in the *Vibrionaceae*. Appl Environ Microbiol.

[9] Gooday GW, Marshall KC (1990). The ecology of chitin degradation. Advances in microbial ecology.

[10] Meibom KL, Li XB, Nielsen AT, Wu C-Y, Roseman S, Schoolnik GK (2004). The *Vibrio cholerae* chitin utilization program. Proc Natl Acad Sci U S A.

[11] Defoirdt T, Darshanee Ruwandeepika HA, Karunasagar I, Boon N, Bossier P (2010). Quorum sensing negatively regulates chitinase in *Vibrio harveyi*. Environ Microbiol Rep.

[12] Li X, Roseman S (2004). The chitinolytic cascade in Vibrios is regulated by chitin oligosaccharides and a two-component chitin catabolic sensor/kinase. Proc Natl Acad Sci U S A.

[13] Orikoshi H, Nakayama S, Miyamoto K, Hanato C, Yasuda M, Inamori Y (2005). Roles of four chitinases (ChiA, ChiB, ChiC, and ChiD) in the chitin degradation system of marine bacterium *Alteromonas* sp. strain O-7. Appl Environ Microbiol.

[14] Markov EY, Kulikalova ES, Urbanovich LY, Vishnyakov VS, Balakhonov SV (2015). Chitin and products of its hydrolysis in *Vibrio cholerae* ecology. Biochemistry (Mosc).

[15] Thompson CC, Vicente ACP, Souza RC, Vasconcelos ATR, Vesth T, Alves N (2009). Genomic taxonomy of vibrios. BMC Evol Biol.

[16] Sawabe T, Ogura Y, Matsumura Y, Gao F, Amin A, Mino S, et al. Updating the vibrio clades defined by multilocus sequence phylogeny: proposal of eight new clades, and the description of *Vibrio tritonius* sp. nov. Front Microbiol. 2013;4(414) 10.3389/fmicb.2013.00414 PMC3873509 24409173

[17] Lukjancenko O, Ussery D. *Vibrio* chromosome-specific families. Front Microbiol. 2014;5(73) 10.3389/fmicb.2014.00073 PMC3957060 24672511

[18] Hazen TH, Pan L, Gu JD, Sobecky PA (2010). The contribution of mobile genetic elements to the evolution and ecology of *Vibrios*. FEMS Microbiol Ecol.

[19] Boucher Y, Cordero OX, Takemura A, Hunt DE, Schliep K, Bapteste E, et al. Local mobile gene pools rapidly cross species boundaries to create endemicity within global *Vibrio cholerae* populations. MBio. 2011;2(2) 10.1128/mBio.00335-10 PMC3073641 21486909

[20] Mardis E, McPherson J, Martienssen R, Wilson RK, McCombie WR (2002). What is finished, and why does it matter. Genome Res.

[21] Marmur J (1961). A procedure for the isolation of deoxyribonucleic acid from micro-organisms. J Mol Biol.

[22] Koren S, Walenz BP, Berlin K, Miller JR, Bergman NH, Phillippy AM. Canu: scalable and accurate long-read assembly via adaptive k-mer weighting and repeat separation. Genome Res. 2017;27(4) 10.1101/gr.215087.116 PMC5411767 28298431

[23] Walker BJ, Abeel T, Shea T, Priest M, Abouelliel A, Sakthikumar S (2014). Pilon: an integrated tool for comprehensive microbial variant detection and genome assembly improvement. PLoS One.

[24] Hunt M, Kikuchi T, Sanders M, Newbold C, Berriman M, Otto TD (2013). REAPR: a universal tool for genome assembly evaluation. Genome Biol.

[25] Brettin T, Davis JJ, Disz T, Edwards RA, Gerdes S, Olsen GJ (2015). RASTtk: a modular and extensible implementation of the RAST algorithm for building custom annotation pipelines and annotating batches of genomes. Sci Rep.

[26] Schattner P, Brooks AN, Lowe TM (2005). The tRNAscan-SE, snoscan and snoGPS web servers for the detection of tRNAs and snoRNAs. Nucleic Acids Res.

[27] Lagesen K, Hallin P, Rødland EA, Stærfeldt H-H, Rognes T, Ussery DW (2007). RNAmmer: consistent and rapid annotation of ribosomal RNA genes. Nucleic Acids Res.

[28] Grissa I, Vergnaud G, Pourcel C (2007). CRISPRFinder: a web tool to identify clustered regularly interspaced short palindromic repeats. Nucleic Acids Res.

[29] Urbanczyk H, Ast JC, Higgins MJ, Carson J, Dunlap PV (2007). Reclassification of *Vibrio fischeri*, *Vibrio logei*, *Vibrio salmonicida* and *Vibrio wodanis* as *Aliivibrio fischeri* gen. nov., comb. nov., *Aliivibrio logei* comb. nov., *Aliivibrio salmonicida* comb. nov. and *Aliivibrio wodanis* comb. nov. Int J Syst Evol Microbiol.

[30] Gauthier G, Lafay B, Ruimy R, Breittmayer V, Nicolas JL, Gauthier M (1995). Small-subunit rRNA sequences and whole DNA relatedness concur for the reassignment of *Pasteurella piscicida* (Snieszko et al.) Janssen and Surgalla to the genus *Photobacterium* as *Photobacterium damsela* subsp. *piscicida* comb. nov. Int J Syst Evol Microbiol.

[31] Contreras-Moreira B, Vinuesa P (2013). GET_HOMOLOGUES, a versatile software package for scalable and robust microbial pangenome analysis. Appl Environ Microbiol.

[32] Kristensen DM, Kannan L, Coleman MK, Wolf YI, Sorokin A, Koonin EV (2010). A low-polynomial algorithm for assembling clusters of orthologous groups from intergenomic symmetric best matches. Bioinformatics.

[33] Li L, Stoeckert CJ, Roos DS (2003). OrthoMCL: identification of ortholog groups for eukaryotic genomes. Genome Res.

[34] Katoh K, Standley DM (2013). MAFFT multiple sequence alignment software version 7: improvements in performance and usability. Mol Biol Evol.

[35] Capella-Gutiérrez S, Silla-Martínez JM (2009). Gabaldón T: trimAl: a tool for automated alignment trimming in large-scale phylogenetic analyses. Bioinformatics.

[36] Stamatakis A (2014). RAxML version 8: a tool for phylogenetic analysis and post-analysis of large phylogenies. Bioinformatics.

[37] Abascal F, Zardoya R, Posada D (2005). ProtTest: selection of best-fit models of protein evolution. Bioinformatics.

[38] Huson DH, Bryant D (2006). Application of phylogenetic networks in evolutionary studies. Mol Biol Evol.

[39] Sanderson MJ (2002). Estimating absolute rates of molecular evolution and divergence times: a penalized likelihood approach. Mol Biol Evol.

[40] Sanderson MJ (2003). r8s: inferring absolute rates of molecular evolution and divergence times in the absence of a molecular clock. Bioinformatics.

[41] Falcon LI, Magallon S, Castillo A (2010). Dating the cyanobacterial ancestor of the chloroplast. ISME J.

[42] Bergthorsson U, Ochman H (1998). Distribution of chromosome length variation in natural isolates of *Escherichia coli*. Mol Biol Evol.

[43] Csűös M (2010). Count: evolutionary analysis of phylogenetic profiles with parsimony and likelihood. Bioinformatics.

[44] Waack S, Keller O, Asper R, Brodag T, Damm C, Fricke WF (2006). Score-based prediction of genomic islands in prokaryotic genomes using hidden Markov models. BMC Bioinformatics.

[45] Goris J, Konstantinidis KT, Klappenbach JA, Coenye T, Vandamme P, Tiedje JM (2007). DNA–DNA hybridization values and their relationship to whole-genome sequence similarities. Int J Syst Evol Microbiol.

[46] Kolde R. pheatmap: Pretty Heatmaps: R package version 1.0.8. Journal 2015, https://cran.r-project.org/web/packages/pheatmap/index.html.

[47] Saitou N, Nei M (1987). The neighbor-joining method: a new method for reconstructing phylogenetic trees. Mol Biol Evol.

[48] Kumar S, Stecher G, Tamura K (2016). MEGA7: molecular evolutionary genetics analysis version 7.0 for bigger datasets. Mol Biol Evol.

[49] Yang Z (2007). PAML 4: phylogenetic analysis by maximum likelihood. Mol Biol Evol.

[50] Barberán A, Casamayor EO (2010). Global phylogenetic community structure and β-diversity patterns in surface bacterioplankton metacommunities. Aquat Microb Ecol.

[51] Stern A, Sorek R (2011). The phage-host arms race: shaping the evolution of microbes. BioEssays.

[52] Tettelin H, Riley D, Cattuto C, Medini D (2008). Comparative genomics: the bacterial pan-genome. Curr Opin Microbiol.

[53] Dorsch M, Lane D, Stackebrandt E (1992). Towards a phylogeny of the genus *Vibrio* based on 16S rRNA sequences. Int J Syst Evol Microbiol.

[54] Sawabe T, Kita-Tsukamoto K, Thompson FL (2007). Inferring the evolutionary history of vibrios by means of multilocus sequence analysis. J Bacteriol.

[55] Konstantinidis KT, Tiedje JM (2005). Genomic insights that advance the species definition for prokaryotes. Proc Natl Acad Sci U S A.

[56] Kim M, Oh H-S, Park S-C, Chun J (2014). Towards a taxonomic coherence between average nucleotide identity and 16S rRNA gene sequence similarity for species demarcation of prokaryotes. Int J Syst Evol Microbiol.

[57] Ochman H, Lawrence JG, Groisman EA (2000). Lateral gene transfer and the nature of bacterial innovation. Nature.

[58] Svitil AL, Chadhain S, Moore JA, Kirchman DL (1997). Chitin degradation proteins produced by the marine bacterium *Vibrio harveyi* growing on different forms of chitin. Appl Environ Microbiol.

[59] Pichyangkura R, Kudan S, Kuttiyawong K, Sukwattanasinitt M, Aiba S-I (2002). Quantitative production of 2-acetamido-2-deoxy-D-glucose from crystalline chitin by bacterial chitinase. Carbohydr Res.

[60] Ohno T, Armand S, Hata T, Nikaidou N, Henrissat B, Mitsutomi M (1996). A modular family 19 chitinase found in the prokaryotic organism *Streptomyces griseus* HUT 6037. J Bacteriol.

[61] Thompson JR, Randa MA, Marcelino LA, Tomita-Mitchell A, Lim E, Polz MF (2004). Diversity and dynamics of a North Atlantic coastal *Vibrio* community. Appl Environ Microbiol.

[62] Constantin de Magny G, Hasan N, Roche B (2014). How community ecology can improve our understanding of cholera dynamics. Front Microbiol.

[63] Reno ML, Held NL, Fields CJ, Burke PV, Whitaker RJ (2009). Biogeography of the Sulfolobus islandicus pan-genome. Proc Natl Acad Sci U S A.

[64] Qin Q-L, Xie B-B, Yu Y, Shu Y-L, Rong J-C, Zhang Y-J (2014). Comparative genomics of the marine bacterial genus *Glaciecola* reveals the high degree of genomic diversity and genomic characteristic for cold adaptation. Environ Microbiol.

[65] Giovannoni SJ, Tripp HJ, Givan S, Podar M, Vergin KL, Baptista D (2005). Genome streamlining in a cosmopolitan oceanic bacterium. Science.

[66] Partensky F, Garczarek L (2010). Prochlorococcus: advantages and limits of minimalism. Annu Rev Mar Sci.

[67] Konstantinidis KT, Serres MH, Romine MF, Rodrigues JLM, Auchtung J, McCue L-A (2009). Comparative systems biology across an evolutionary gradient within the *Shewanella* genus. Proc Natl Acad Sci U S A.

[68] Touchon M, Hoede C, Tenaillon O, Barbe V, Baeriswyl S, Bidet P (2009). Organised genome dynamics in the *Escherichia coli* species results in highly diverse adaptive paths. PLoS Genet.

[69] Tanner AR, Fuchs D, Winkelmann IE, Gilbert MT, Pankey MS, Ribeiro AM et al. Molecular clocks indicate turnover and diversification of modern coleoid cephalopods during the Mesozoic Marine Revolution. Proc Biol Sci. 2017;284(1850):20162818. 10.1098/rspb.2016.2818 PMC5360930 28250188

[70] Near TJ, Eytan RI, Dornburg A, Kuhn KL, Moore JA, Davis MP (2012). Resolution of ray-finned fish phylogeny and timing of diversification. Proc Natl Acad Sci U S A.

[71] Ruby EG, McFall-Ngai MJ (1999). Oxygen-utilizing reactions and symbiotic colonization of the squid light organ by *Vibrio fischeri*. Trends Microbiol.

[72] Konstantinidis KT, Tiedje JM (2004). Trends between gene content and genome size in prokaryotic species with larger genomes. Proc Natl Acad Sci U S A.

[73] Eagon RG (1962). *Pseudomonas natriegens*, a marine bacterium with a generation time of less than 10 minutes. J Bacteriol.

[74] Klappenbach JA, Dunbar JM, Schmidt TM (2000). rRNA operon copy number reflects ecological strategies of bacteria. Appl Environ Microbiol.

[75] Flemming H-C, Wingender J (2010). The biofilm matrix. Nat Rev Microbiol.

[76] Karlsson M, Stenlid J, Pontarotti P (2009). Comparative evolutionary histories of fungal chitinases. Evolutionary biology: concept, modeling, and application.

[77] Keyhani NO, Roseman S (1999). Physiological aspects of chitin catabolism in marine bacteria. Biochim Biophys Acta-Gen Subj.

[78] Qin Q-L, Li Y, Zhang Y-J, Zhou Z-M, Zhang W-X, Chen X-L (2011). Comparative genomics reveals a deep-sea sediment-adapted life style of *Pseudoalteromonas* sp. SM9913. ISME J.

[79] Nei M, Rooney AP (2005). Concerted and birth-and-death evolution of multigene families. Annu Rev Genet.

[80] Meibom KL, Blokesch M, Dolganov NA, Wu C-Y, Schoolnik GK (2005). Chitin induces natural competence in *Vibrio cholerae*. Science.

