# Supplementary material for: Comparative genomic analysis reveals the evolution and environmental adaptation strategies of vibrios
Source: BMC Genomics. 2018 Feb 13;19:135. doi: 10.1186/s12864-018-4531-2 (PMC5809883; doi:10.1186/s12864-018-4531-2)
Supplement: Supplementary file 1 — Table S1. General features of the 20 complete genomes of Vibrio species analyzed. (DOCX 48 kb) [file 12864_2018_4531_MOESM1_ESM.docx]

**Additional file 1: Table S1** General features of the 19 complete genomes of *Vibrio* species analyzed.

| Strains | Size (Mb) | ORF No. | Orthologous  cluster No. | G+C Content (%) | 16S rRNA Gene Copies | tRNA Gene No. | R-M system^b^ | CRISPR presence | GenBank Accession | Isolation source |
| --- | --- | --- | --- | --- | --- | --- | --- | --- | --- | --- |
| *V. alginolyticus* ATCC 17749 | 5.15 | 4835 | 4677 | 44.7 | 11 | 105 | I | no | CP006718-19 | Horse mackerel |
| *V. anguillarum* 90-11-286 | 4.34 | 4060 | 3825 | 44.4 | 10 | 106 | I | yes | CP011460-61 | Fish farm water |
| *V. breoganii* FF50 | 4.49 | 4255 | 4027 | 45.2 | 9 | 102 | - | no | CP016177-79 | Seawater |
| *V. campbellii* ATCC BAA-1116 | 6.03 | 5920 | 5170 | 45.4 | 10 | 96 | I | yes | CP006605-07 | Seawater |
| *V. cholerae* KW3 | 4.09 | 3807 | 3693 | 47.5 | 7 | 81 | I, II | no | CP006947-48 | Human |
| *V. coralliilyticus* RE98 | 6.04 | 5830 | 5629 | 45.5 | 11 | 116 | I, III | no | CP009617-20 | Shellfish |
| *V. damselae* KC-Na-1 | 4.54 | 3811 | 3567 | 40.9 | 15 | 193 | I | yes | CP021151-56 | Finless porpoise |
| *V. fischeri* ES114 | 4.27 | 3899 | 3756 | 38.3 | 12 | 119 | I | no | CP000020-22 | Squid |
| *V. fluvialis* ATCC 33809 | 4.83 | 4542 | 4389 | 49.9 | 10 | 108 | I | yes | CP014034-35 | Human |
| *V. furnissii* NCTC 11218 | 4.92 | 4749 | 4553 | 50.7 | 7 | 100 | I | no | CP002377-78 | Estuary |
| *V. harveyi* ATCC 43516 | 6.04 | 5617 | 5372 | 44.9 | 12 | 133 | I | yes | CP014038-39 | Shark |
| ***V. mediterranei* QT6D1^a^** | 5.81 | 5381 | 3947 | 44.2 | 11 | 114 | I | yes | CP018308-09 | Seawater |
| *V. mimicus* ATCC 33654 | 4.44 | 4186 | 5458 | 46.4 | 9 | 101 | I | no | CP014042-43 | Lake water |
| *V. nigripulchritudo* SFn1 | 6.32 | 5829 | 4671 | 45.7 | 8 | 103 | I | no | FO203526-27 | Shrimp |
| *V. parahaemolyticus* ATCC 17802 | 5.15 | 4808 | 4645 | 45.3 | 14 | 134 | I | no | CP014046-47 | Human |
| ***V. rotiferianus* B64D1^a^** | 5.28 | 4816 | 5017 | 44.8 | 13 | 133 | I, II | no | CP018311-12 | Seawater |
| *V. tasmaniensis* LGP32 | 4.97 | 4480 | 4323 | 43.9 | 8 | 114 | I | yes | FM954972-73 | Oyster |
| *V. tritonius* JCM 16456 | 5.22 | 4831 | 4525 | 43.9 | 11 | 118 | - | yes | AP014635-36 | Sea hare |
| *V. tubiashii* ATCC 19109 | 5.54 | 5203 | 5002 | 45.0 | 10 | 117 | I, II | no | CP009354-59 | Hard clam |
| *V. vulnificus* FORC 017 | 5.23 | 4911 | 4686 | 46.6 | 11 | 115 | I, II | no | CP012739-41 | Human |

^a^ Genomes of these strains were sequenced in this study.

^b^ I = Type I Restriction-Modification (R-M) system, II = Type II R-M system, III = Type III R-M system, - = no R-M system.
