# Supplementary material for: Comparative genomic analysis reveals the evolution and environmental adaptation strategies of vibrios
Source: BMC Genomics. 2018 Feb 13;19:135. doi: 10.1186/s12864-018-4531-2 (PMC5809883; doi:10.1186/s12864-018-4531-2)
Supplement: Supplementary file 2 — Table S2. General features of the 13 marine bacteria complete genomes used to compare with Vibrio. (DOCX 34 kb) [file 12864_2018_4531_MOESM2_ESM.docx]

**Additional file 2: Table S2** General features of the 13 marine bacteria complete genomes used to compare with *Vibrio*.

| **Bacterial genomes** | **Genome Size (Mb)** | **G+C content (%)** | **16S rRNA gene copies** | **HGT genes (%)** | **Chitinase No.** | **GenBank Accession** |
| --- | --- | --- | --- | --- | --- | --- |
| ***Cyanobacteria*** |  |  |  |  |  |  |
| *Prochlorococcus marinus* CCMP1375^T^ | 1.75 | 40.6 | 1 | 0.85 | 0 | AE017126 |
| *Synechococcus elongatus* PCC 6301 | 2.70 | 55.5 | 2 | 2.69 | 0 | AP008231 |
| ***Gammaproteobacteria*** |  |  |  |  |  |  |
| *Alteromonas macleodii* ATCC 27126^T^ | 4.65 | 44.7 | 5 | 0.77 | 0 | CP003841 |
| *Marinobacter hydrocarbonoclasticus* ATCC 49840^T^ | 3.99 | 57.4 | 3 | 9.96 | 0 | FO203363 |
| *Pseudoalteromonas phenolica* KCTC 12086^T^ | 4.87 | 40.6 | 9 | 1.78 | 8 | CP013187-88 |
| *Shewanella denitrificans* OS217^T^ | 4.55 | 45.1 | 8 | 3.73 | 2 | CP000302 |
| ***Alphaproteobacteria*** |  |  |  |  |  |  |
| *Erythrobacter atlanticus* s21-N3^T^ | 3.23 | 58.3 | 1 | 3.83 | 0 | CP011310, CP015441 |
| *Rhodobacter sphaeroides* ATCC 17025 | 4.56 | 68.2 | 4 | 9.25 | 0 | CP000661-66 |
| *Ruegeria pomeroyi* DSS-3 | 4.60 | 64.1 | 3 | 4.56 | 0 | CP000031-32 |
| *Candidatus Pelagibacter ubique* HTCC1062 | 1.31 | 29.7 | 1 | 0.07 | 0 | CP000084 |
| ***Flavobacteriia*** |  |  |  |  |  |  |
| *Croceibacter atlanticus* HTCC2559^T^ | 2.95 | 33.9 | 2 | 1.50 | 0 | CP002046 |
| *Owenweeksia hongkongensis* DSM 17368^T^ | 4.00 | 40.2 | 2 | 0.14 | 0 | CP003156 |
| *Nonlabens dokdonensis* DSW-6^T^ | 3.91 | 35.3 | 2 | 0.00 | 0 | CP001397 |
