# Supplementary material for: Comparative genomic analysis reveals the evolution and environmental adaptation strategies of vibrios
Source: BMC Genomics. 2018 Feb 13;19:135. doi: 10.1186/s12864-018-4531-2 (PMC5809883; doi:10.1186/s12864-018-4531-2)
Supplement: Supplementary file 3 — Figure S1. Pan and core plot of the 20 Vibrio species with complete genomes. Accumulation curves for (A) the number of genes in common or (B) total number of genes are plotted. The grey dots represent 10 different random input sequences of genomes, and the extrapolated limiting value for core genes is shown as a dashed line. Equations which can be used to fit the curves are shown above the plot respectively. (PDF 1023 kb) [file 12864_2018_4531_MOESM3_ESM.pdf]

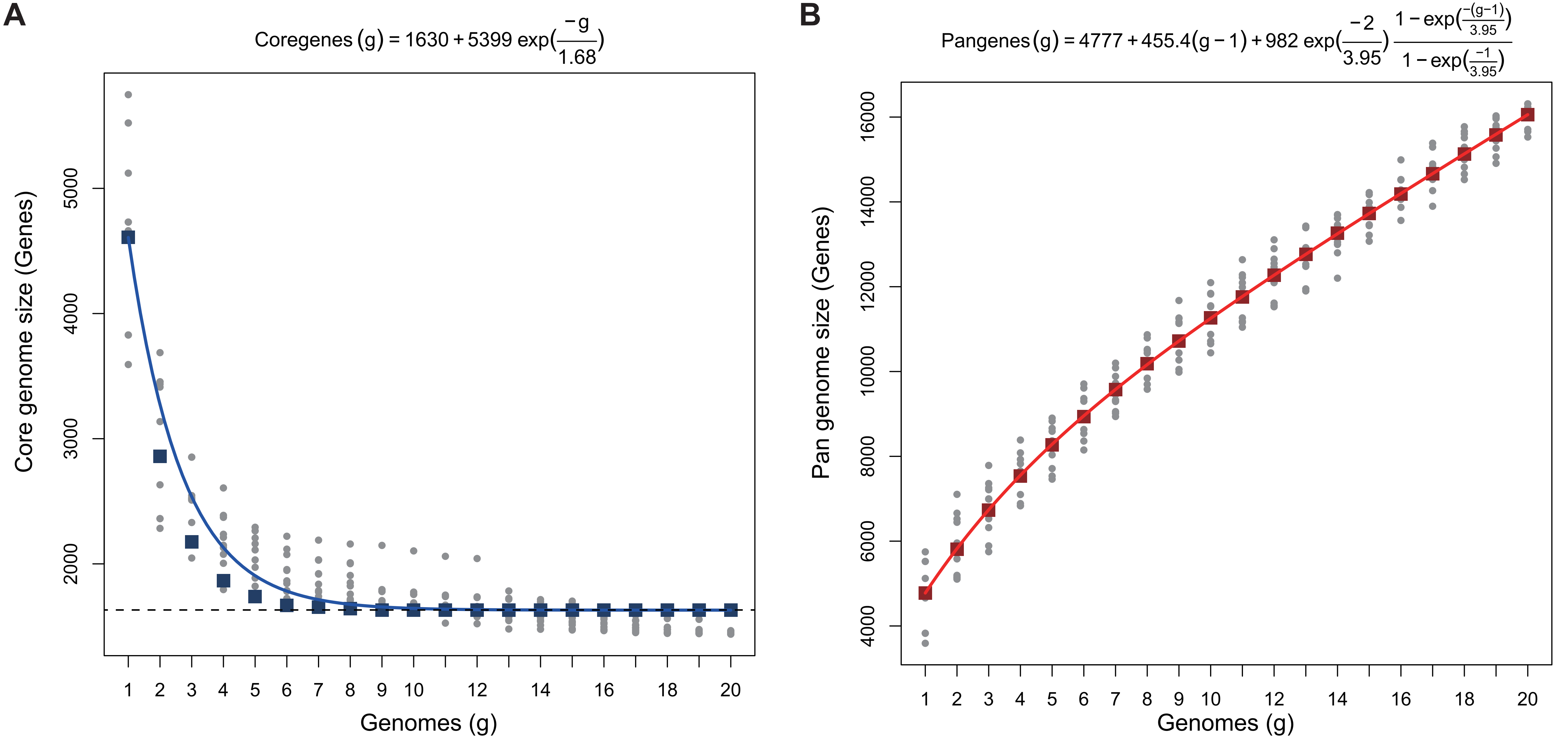
A
Coregenes (g) = 1630 + 5399 exp(−g/1.68)
Core genome size (Genes)
5000
4000
3000
2000
1
2
3
4
5
6
7
8
9
10
12
14
16
18
20
Genomes (g)
B
Pangenes (g) = 4777 + 455.4(g − 1) + 982 exp(−2/3.95) (1 − exp(−(g−1)/3.95)) / (1 − exp(−1/3.95))
Pan genome size (Genes)
16000
14000
12000
10000
8000
6000
4000
1
2
3
4
5
6
7
8
9
10
12
14
16
18
20
Genomes (g)
