# Supplementary figures and images for: Comparative genomic analysis reveals the evolution and environmental adaptation strategies of vibrios
Source: BMC Genomics. 2018 Feb 13;19:135. doi: 10.1186/s12864-018-4531-2 (PMC5809883; doi:10.1186/s12864-018-4531-2)

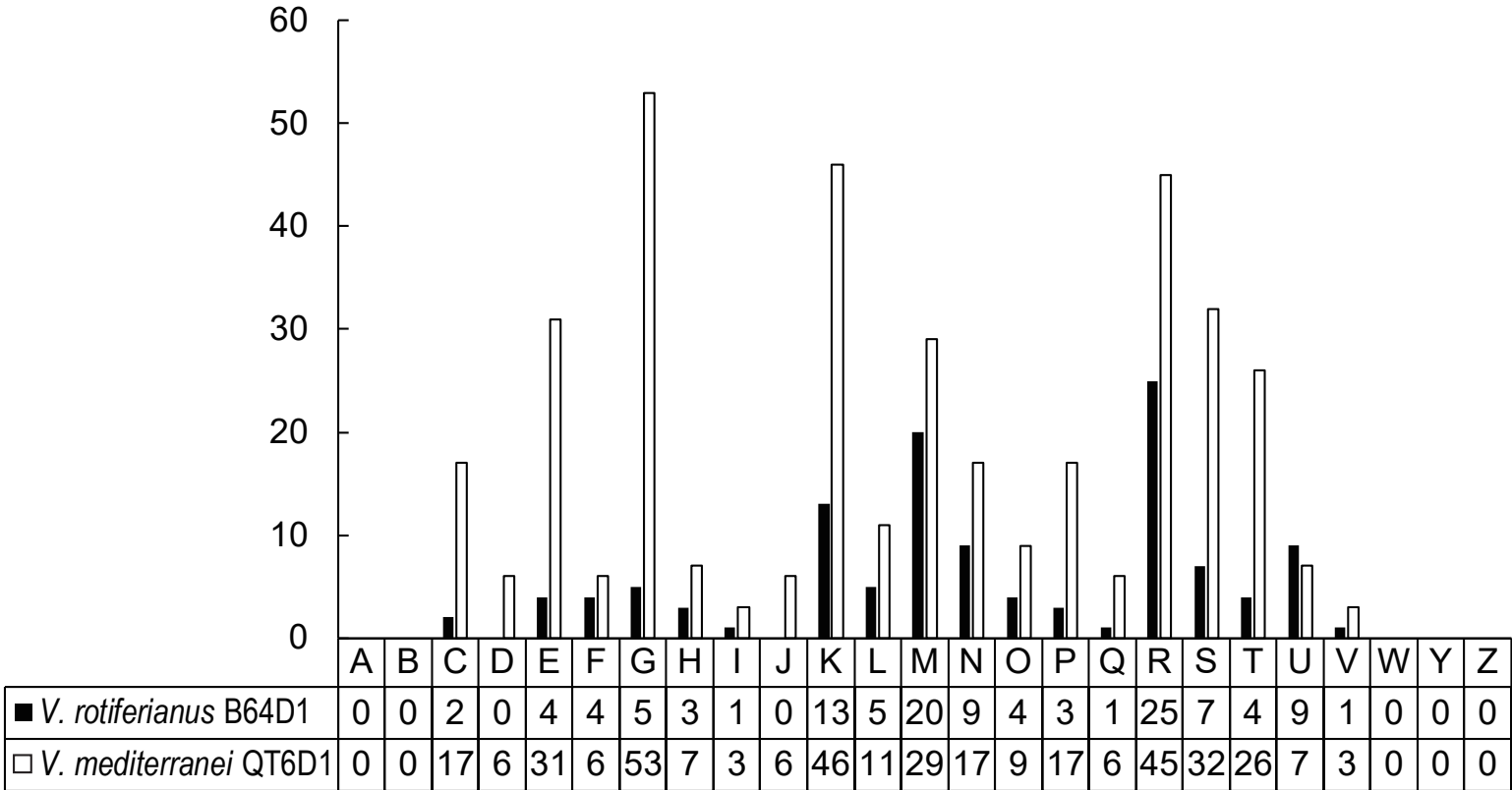

Supplement: Supplementary file 4 — Figure S2. COGs classification of the specific genomes of V. rotiferianus B64D1 and V. mediterranei QT6D1. Designations of functional categories: [A] RNA processing and modification, [B] chromatin structure and dynamics, [C] energy production and conversion, [D] cell cycle control and mitosis, [E] amino acid metabolism and transport, [F] nucleotide metabolism and transport, [G] carbohydrate metabolism and transport, [H] coenzyme metabolism, [I] lipid metabolism, [J] translation, [K] transcription, [L] replication and repair, [M] cell wall/membrane/envelope biogenesis, [N] Cell motility, [O] post-translational modification, protein turnover, chaperone functions, [P] Inorganic ion transport and metabolism, [Q] secondary metabolites biosynthesis, transport and catabolism, [R] general functional prediction only, [S] function unknown, [T] signal transduction, [U] intracellular trafficking and secretion, [V] Defense mechanisms, [W] Extracellular structures, [Y] nuclear structure, [Z] cytoskeleton. (PDF 822 kb) [file 12864_2018_4531_MOESM4_ESM.pdf]

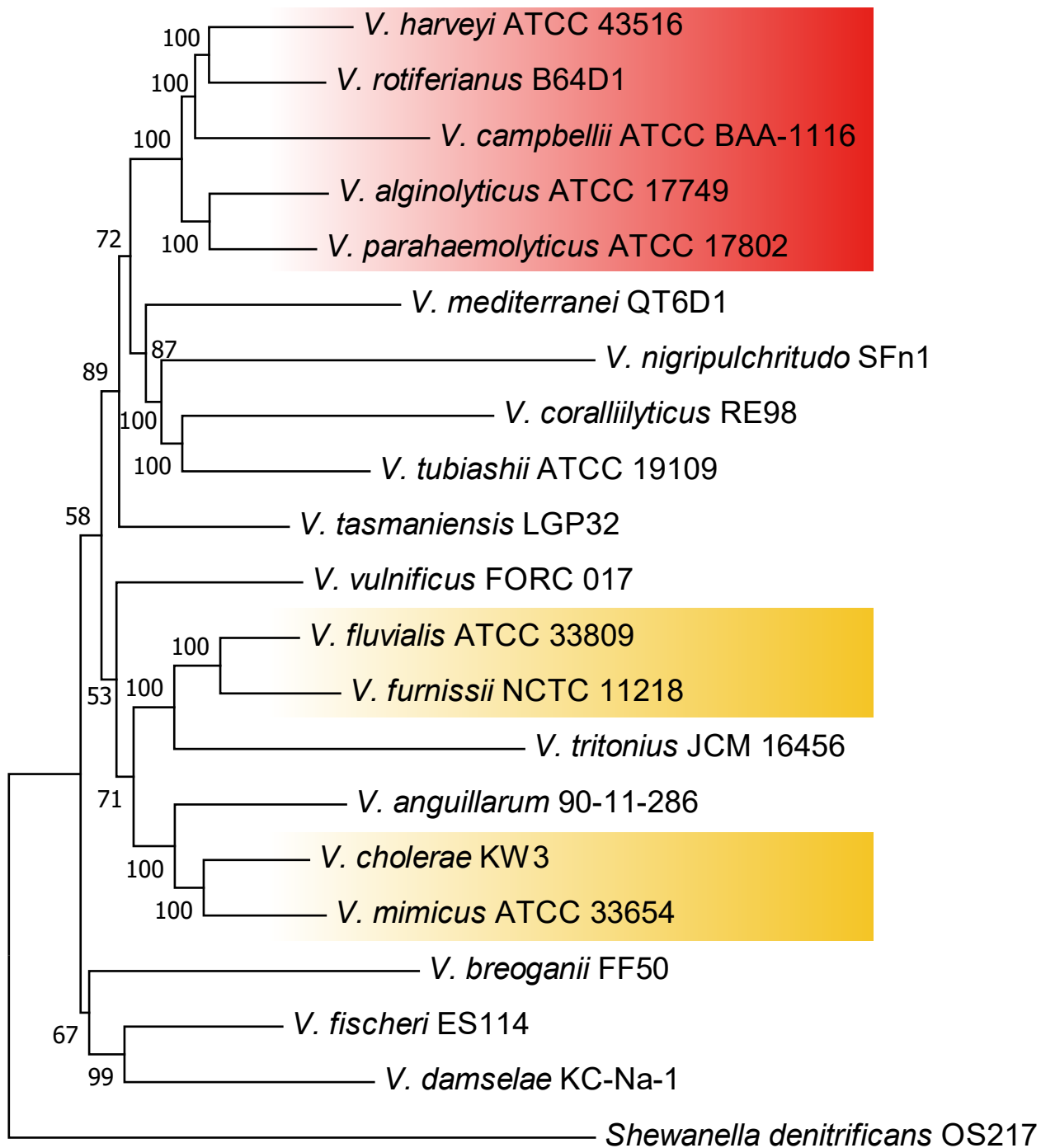

0.02

Supplement: Supplementary file 5 — Figure S3. Pan genome tree. The tree was created based on the presence or absence of gene clusters in the 20 complete Vibrio genomes. The number at each node denotes the bootstrap value based on 1000 replicates. The color red and yellow are corresponding to the core genome tree, suggesting the discrepancy between core and pan genome trees. (PDF 194 kb) [file 12864_2018_4531_MOESM5_ESM.pdf]

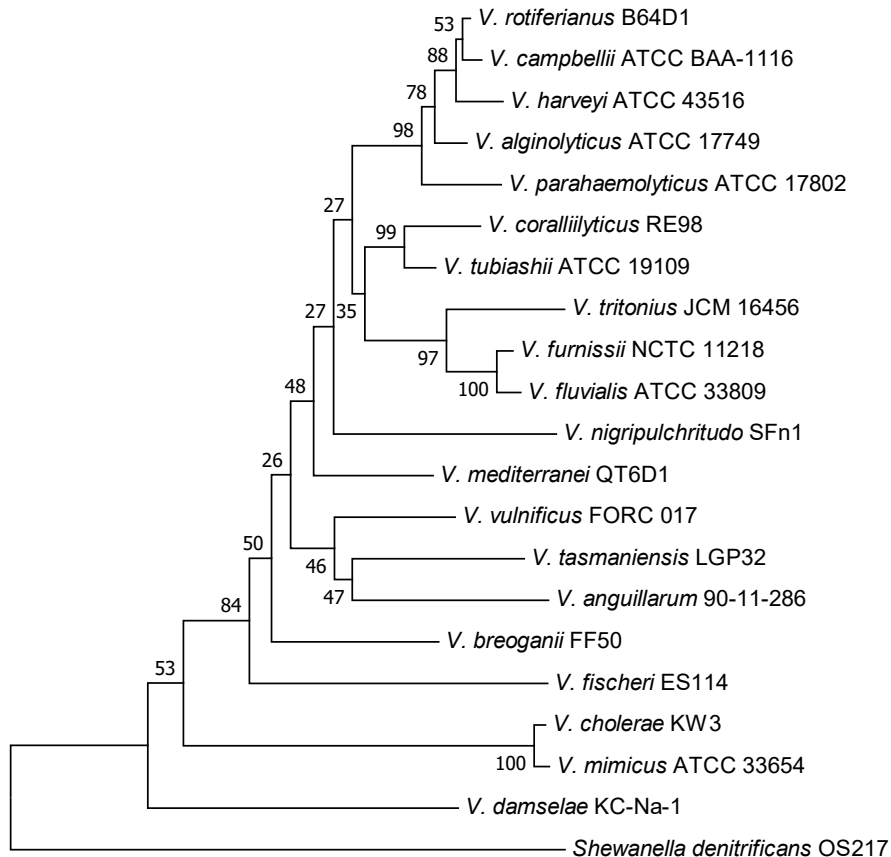

0.01

Supplement: Supplementary file 6 — Figure S4. Neighbor-joining phylogenetic tree of the 20 vibrios genomes based on 16S rRNA gene. The number at each node denotes the bootstrap value based on 1000 replicates. S. denitrificans OS217 was used as the outgroup. Bar, 0.01 substitutions per site. (PDF 186 kb) [file 12864_2018_4531_MOESM6_ESM.pdf]

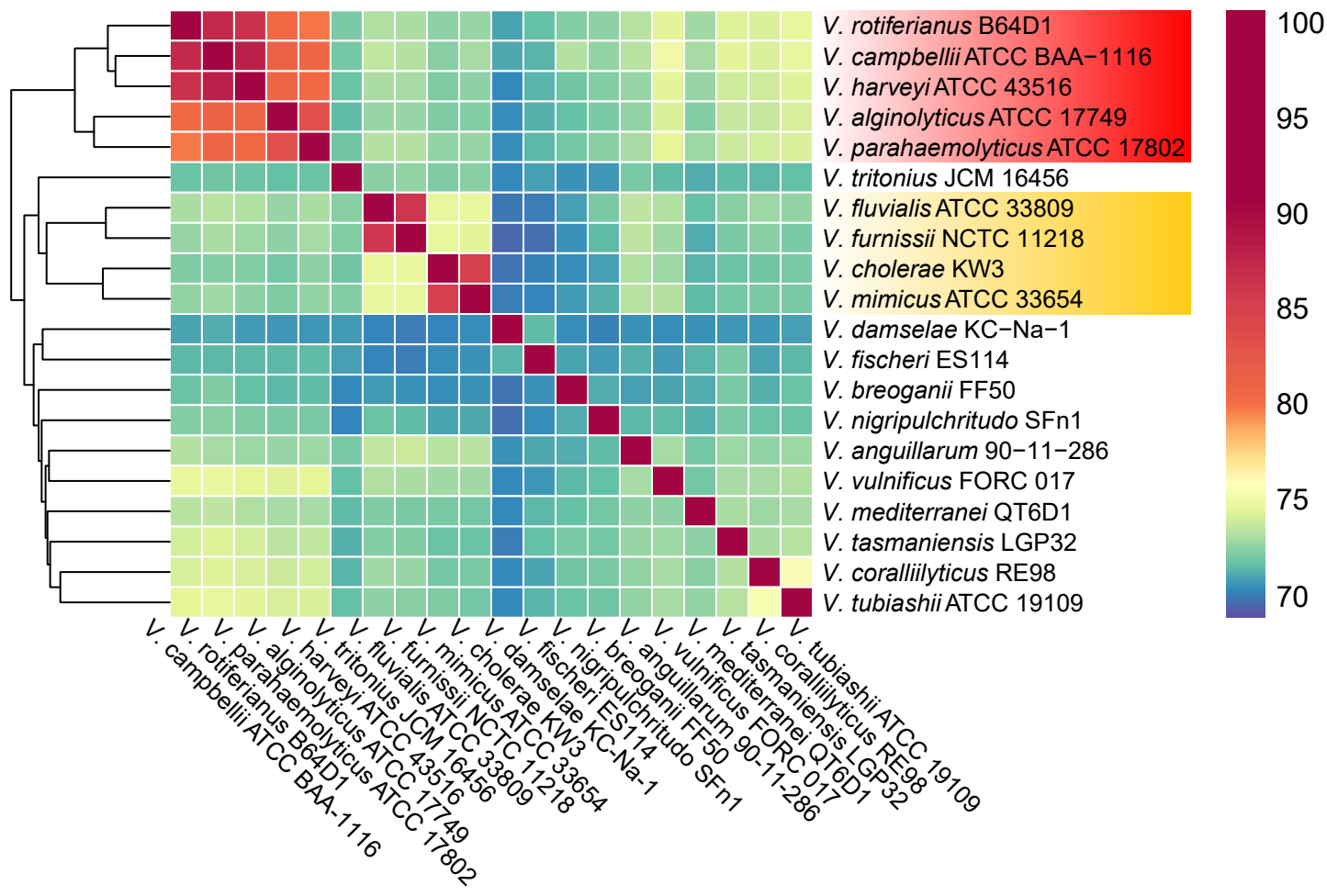

Supplement: Supplementary file 7 — Figure S5. Heatmap presentation of pairwise average nucleotide identity of the 19 Vibrio species with complete genomes. The genomes are hierarchical clustered according to the values of rows. The clusters present in blue and orange are corresponding to the core genome tree. (PDF 212 kb) [file 12864_2018_4531_MOESM7_ESM.pdf]

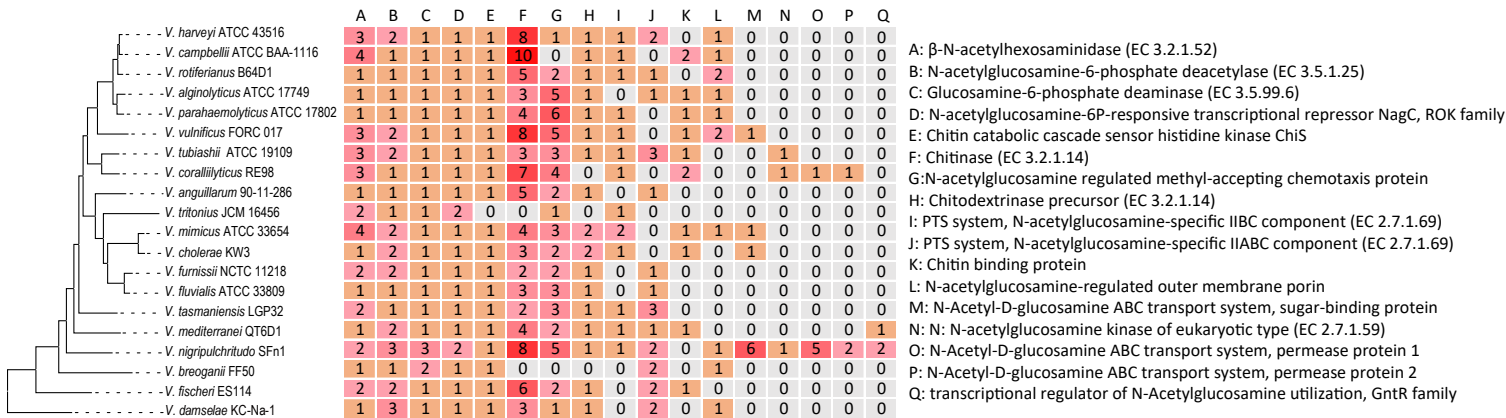

Supplement: Supplementary file 8 — Figure S6. Genes related to the chitin-degrading process in 20 vibrios with complete genomes. Each column indicates a chitin metabolism-related gene family, with the family name indicating the predicted function. The number in the box indicates the copy number of that gene family in the corresponding genome. (PDF 282 kb) [file 12864_2018_4531_MOESM8_ESM.pdf]
